# Evaluation of FGD3 Expression in Advanced Prostate Cancer

**DOI:** 10.3390/biomedicines14102254

**Published:** 2026-10-06

**Authors:** Bartosz Malinowski, Paulina Antosik, Layla Settaf-Cherif, Dariusz Grzanka, Michał Wiciński

**Affiliations:** 1 Department of Pharmacology and Therapeutics, Faculty of Medicine, Collegium Medicum in Bydgoszcz, Nicolaus Copernicus University in Toruń, 85-094 Bydgoszcz, Poland; 2 Department of Dermatology and Venerology, Faculty of Medicine, Collegium Medicum in Bydgoszcz, Nicolaus Copernicus University in Toruń, 85-094 Bydgoszcz, Poland; paulina.antosik@cm.umk.pl; 3 Department of Urology and Andrology, Ludwik Rydygier Collegium Medicum, Nicolaus Copernicus University in Toruń, 85-094 Bydgoszcz, Poland; layla.cherif@doktorant.umk.pl; 4 Department of Clinical Pathomorphology, Faculty of Medicine, Collegium Medicum in Bydgoszcz, Nicolaus Copernicus University in Toruń, 85-094 Bydgoszcz, Poland; d_grzanka@cm.umk.pl

**Keywords:** FGD3, prostate cancer, RhoGEF, expression, survival, diagnostic factor

## Abstract

**Background/Objectives**: Prostate cancer (PCa) is a leading health issue among men, with its impact varying significantly from one individual to another. The Faciogenital Dysplasia 3 (FGD3) has been described as a strong independent prognostic marker in breast cancer. The aim of our study was to investigate the possible impact of FGD3 expression on PCa progression and aggressiveness, as well as its correlation with clinical outcomes. **Methods**: Expression of FGD3 has been evaluated by immunohistochemical methods. The study cohort was enriched for cases with poorer prognosis and locally advanced disease, with complete clinicopathological data available, including age, Grade Group (ISUP), pathological T stage (pT), nodal status (pN), Gleason score, prostate-specific antigen (PSA) levels, and TNM stage. **Results**: Analysis of FGD3 immunoexpression revealed significant differences between control prostate tissue, primary prostate cancer tissue, and lymph node metastases. Pairwise comparisons showed significantly lower FGD3 expression in prostate cancer tissues compared with control tissues (*p* = 0.0483). Moreover, FGD3 expression was significantly reduced in lymph node metastases compared with both control tissues (*p* < 0.0001) and primary prostate cancer tissues (*p* < 0.0001). **Conclusions**: FGD3 was determined for the first time in patients with advanced prostate cancer. Our findings showed that reduced FGD3 expression has adverse prognostic significance in PCa. Further investigation is needed to confirm its possible use in daily clinical practice.

## 1. Introduction

Prostate adenocarcinoma (PRAD) is the most common form of cancer found within the prostate gland and is globally recognized as a significant health concern. This malignancy demonstrates a broad spectrum of clinical behaviors, from indolent growth to aggressive metastatic spread [1]. Notably, the incidence and mortality rates associated with this disease vary significantly across different racial and ethnic demographics [2,3]. Molecular discrepancies may affect the varied incidence rates and clinical consequences [4].

This variation in disease manifestation and outcomes may be attributed to intricate biological factors. The Faciogenital Dysplasia 3 (FGD3), biochemically part of the FGD family of RhoGEFs, plays an important role in cancer development. It is responsible for the regulation of the Ras Homolog (Rho) family of GTPases, which are essential for various cellular processes, such as cell morphology, migration, and cell cycle progression [5]. Therefore, FGD-3’s complex role in cancerogenesis continues to be explored.

Elevated FGD-3 mRNA levels have been associated with more favorable outcomes across several cancer types, highlighting FGD3’s potential as a comprehensive prognostic biomarker and offering valuable insights into disease progression and therapeutic approaches [6,7].

In several cancers, higher FGD3 expression has been linked to favorable outcomes. Studies in multiple cancer cohorts, head and neck squamous cell carcinoma, stage II/III colorectal cancer, breast cancer, and lung adenocarcinoma have reported an association between preserved or high FGD3 expression and improved prognosis or less aggressive disease. However, the role of FGD3 in PRAD has not been yet evaluated [8,9,10,11,12].

In our research, we aim to deepen understanding of FGD3’s specific functions in PCa progression, focusing on adenocarcinoma. By investigating the correlation between FGD3 expression and clinicopathological characteristics, including disease severity, we strive to establish its efficacy as a possible prognostic marker and therapeutic target.

## 2. Materials and Methods

### 2.1. Samples

The study was conducted on archival formalin-fixed paraffin-embedded (FFPE) tissue samples obtained from 185 Polish patients diagnosed with PRAD, who underwent radical prostatectomy at the General and Oncological Urology Clinic, Antoni Jurasz University Hospital No. 1 in Bydgoszcz (Poland) between 2016 and 2022. Two pathologists independently confirmed the histopathological diagnoses, selecting only those samples that came from patients who also had primary tumors. The study cohort was enriched for cases with poorer prognosis and locally advanced disease, with complete clinicopathological data available, including age, Grade Group (ISUP), pathological T stage (pT), nodal status (pN), Gleason score, prostate-specific antigen (PSA) levels, and TNM stage. Tumor staging was determined according to the 8th edition of the American Joint Committee on Cancer (AJCC) classification system. The study group included 185 prostate cancer tissue samples.

In comparison, the control group consisted of 30 histologically confirmed adjacent non-cancerous prostate tissue samples obtained from the same radical prostatectomy specimens as the corresponding primary tumors. The absence of carcinoma was confirmed histologically by pathologists. These samples were not derived from healthy donors. The distribution of clinicopathological features of the study group is summarized in Table 1.

Additionally, lymph node metastasis (LNM) FFPE samples were analyzed in a subgroup of 27 patients classified as pN1. These samples were derived from the same patients as those with primary tumors and included only lymph nodes excised during radical prostatectomy.

Overall Survival (OS) was defined as the time from diagnosis to death or last follow-up. The follow-up period ranged from 1 to 120 months, and data collection was completed on 15 February 2026.

This research received ethical approval from the Ethics Committee of Nicolaus Copernicus University in Torun, Ludwik Rydygier Collegium Medicum in Bydgoszcz (approval number 248/2019).

### 2.2. Immunohistochemistry

#### 2.2.1. Tissue Macroarrays (TMAs)

Immunohistochemical staining was performed on TMAs prepared from regions representative of the tumor (containing at least 80% tumor cells). Representative areas in tumor tissue samples were identified microscopically using archival hematoxylin and eosin-stained tissue slides (H&E). Subsequently, cylindrical 4 mm tissue cores FFPE were removed from each donor block and placed in the recipient blocks using hand punches (PFM Medical, Cologne, Germany). Each recipient block contained fifteen distinct tissue fragments sourced from donor paraffin blocks, in most cases, three cores were taken from one patient from different tumor areas. A single tissue core was used in the control group and in blocks with LNM. The analysis of LNM is specifically conducted on samples derived from the same patients as those with primary tumors. The analysis only considered the lymph nodes excised during prostate resection. The prepared TMAs were routinely cut into 4 µm thick paraffin sections using a rotary microtome (Accu-Cut^®^ SRMTM200, Sakura Finetek, Torrance, CA, USA), applied to special slides with high adhesion (Superfrost Plus; Menzel Gläser, Thermo Scientific, Braunschweig, Germany), and dried at 60 °C for 1 h.

#### 2.2.2. Immunohistochemical Staining

To evaluate FGD3 protein expression in prostate cancer tissue, adjacent non-cancerous prostate tissue, and lymph nodes with metastatic involvement, immunohistochemical staining was performed using a rabbit polyclonal anti-FGD3 antibody (HPA020963, Sigma-Aldrich, St. Louis, MO, USA) at a dilution of 1:900. The immunohistochemical staining conditions were optimized according to the manufacturer’s recommendations and data available in The Human Protein Atlas. The staining procedure was performed using an automated BenchMark^®^ Ultra system (Ventana Medical Systems, Tucson, AZ, USA), following a previously established protocol. Antigen–antibody complexes were visualized using dedicated detection kits (Roche Diagnostics/Ventana, Medical Systems, Tucson, AZ, USA) according to the manufacturer’s instructions. After completion of the immunohistochemical reaction, the sections were counterstained with hematoxylin (Roche Diagnostics/Ventana Medical Systems, Tuscon, AZ, USA) and treated with bluing reagent (Roche Diagnostics/Ventana Medical Systems, Tucson, AZ, USA). The slides were then dehydrated through a graded ethanol series (80%, 90%, 96%, and 99.8%), cleared in xylene, and mounted with coverslips using Consul Mount Histology medium (Epredia, Menzel Gläser, Germany).

#### 2.2.3. Immunostaining Evaluation

Slides were digitized using a Roche Ventana DP 200 whole-slide imaging scanner (Roche Diagnostics/Ventana Medical Systems, Tucson, AZ, USA) and evaluated by a pathologist (DG). Because FGD3 expression is consistently detected in lymphoid cells, tonsil tissue was used as a positive external control. At the same time, stromal lymphocytes present within the examined specimens served as an internal positive control. Immunohistochemical assessment of FGD3 expression was performed in the cell membrane and cytoplasm of primary prostate tumors, lymph node metastases, and adjacent non-cancerous prostate tissues.

FGD3 protein expression was quantified using the H-score method, which combines staining intensity with the percentage of positively stained cells. Staining intensity was scored as follows: 0, negative; 1, weak; 2, moderate; and 3, strong. The final H-score ranged from 0 to 300 and was calculated as the sum of the products of staining intensity and the corresponding percentage of cells showing that intensity.

FGD3 expression was dichotomized into low- and high-expression groups based on an optimal cut-off value determined using the Evaluate Cutpoints software in R (version R-4.6.1). The optimal cut-off was calculated using the cutp method from the survMisc R package, which fits Cox proportional hazards models across all possible cut-off values and selects the threshold that provides the most significant separation in survival between the two resulting groups based on the log-rank test. This approach allowed patients to be stratified into low- and high-expression groups in a manner that maximized prognostic discrimination while maintaining clinical interpretability. In our cohort, the established cut-off value was 170; therefore, samples with an H-score < 170 were classified as having low FGD3 expression, whereas samples with an H-score ≥ 170 were classified as having high FGD3 expression.

#### 2.2.4. In Silico Analysis TCGA-PRAD Cohort

For external validation, FGD3 mRNA expression was analyzed using RNA-seq transcriptomic data from the TCGA-PRAD cohort, retrieved from the UCSC Xena Browser (http://xena.ucsc.edu; accessed on 13 April 2026). Gene expression profiles were obtained as RSEM expected counts and normalized using the DESeq2 method, which applies the median-of-ratios approach to account for differences in sequencing depth and RNA composition across samples. The original transcriptomic data were aligned using the STAR algorithm and quantified with RSEM.

For survival analysis, 495 patients with available FGD3 expression data and complete overall survival (OS) information, including OS status and OS time, were included in the Kaplan–Meier analysis. In addition, matched normal prostate tissue samples from 51 patients available in the TCGA-PRAD dataset were used to compare FGD3 expression between tumor and non-cancerous tissues. Associations between FGD3 mRNA expression and clinicopathological parameters were assessed in a subset of 490 patients with available clinical and pathological data. For survival-based stratification, an optimal FGD3 mRNA expression cut-off value of 7.566 was established. Samples with expression levels below this threshold were classified as having low FGD3 expression, whereas samples with expression levels equal to or above this threshold were categorized as having high FGD3 expression.

### 2.3. Statistical Analysis

Statistical analyses and graphical presentation of the results were performed using GraphPad Prism software (version 8.0; GraphPad Software, San Diego, CA, USA) and RStudio (version 1.3.1093). The distribution of continuous variables was assessed using the Shapiro–Wilk test. Depending on data distribution and study design, appropriate parametric or non-parametric tests were applied. In the subset of 27 patients for whom matched adjacent non-cancerous prostate tissue, primary prostate cancer tissue, and lymph node metastasis samples were available, differences in FGD3 protein expression among the three tissue types were analyzed using the Friedman test. Post hoc pairwise comparisons were performed using the Wilcoxon matched-pairs signed-rank test with Holm adjustment for multiple comparisons. Kendall’s W was calculated as a measure of effect size for the overall repeated-measures comparison. In the TCGA-PRAD cohort, differences in FGD3 mRNA expression between non-cancerous prostate tissue and prostate cancer tissue samples were assessed using the Mann–Whitney U test. Rank-biserial correlation with a 95% bootstrap confidence interval was calculated as the effect size. Associations between categorized FGD3 expression levels and clinicopathological parameters were evaluated using Pearson’s chi-square test or Fisher’s exact test, as appropriate, according to expected cell frequencies in contingency tables.

For the pT analysis, the Fisher–Freeman–Halton exact test was used due to low expected frequencies in some cells. To account for multiple testing across clinicopathological variables, *p*-values were adjusted using the Benjamini–Hochberg false discovery rate (FDR) procedure, separately within each cohort. Both unadjusted and FDR-adjusted *p* values were reported. Cramér’s V was calculated as a measure of effect size. FGD3 expression was categorized into low- and high-expression groups according to the previously established cut-off value. Overall survival (OS) was analyzed using the Kaplan–Meier method, and differences between survival curves were assessed using the log-rank test. The associations of FGD3 expression and clinicopathological variables with OS were further evaluated using Cox proportional hazards regression models. Both univariable and multivariable analyses were performed, and the results were reported as hazard ratios (HRs) with corresponding 95% confidence intervals (95% CIs).

Variables for the multivariable Cox regression model were selected using a backward stepwise procedure, with variables showing *p* > 0.10 sequentially removed from the model. The proportional hazards assumption was assessed using log-minus-log survival plots and Schoenfeld residuals. No relevant violations of the proportional hazards assumption were detected. All statistical tests were two-sided. A *p*-value < 0.05 was considered statistically significant for analyses not requiring multiplicity adjustment. For analyses involving multiple comparisons, statistical significance was determined based on the corresponding Holm-adjusted or FDR-adjusted *p* value. D’Amico risk classification was assessed using the Evidencio Medical Decision Support tool [13].

### 2.4. Bioinformatics Analyses—FGD3 Interaction Network and Functional Enrichment Analysis

Protein–protein interaction (PPI) network analysis was performed using the STRING database, version 12.0 (accessed on 12 December 2025). The human FGD3 gene was used as the input/query gene. A network comprising FGD3 and 27 co-occurring genes was generated by selecting the first-shell interactors. Only interactions with a minimum required interaction confidence score of 0.4 were retained for visualization and downstream analysis.

Gene association network and functional enrichment analysis were additionally conducted using the GeneMANIA Cytoscape application, version 3.5.3. The evaluation was performed using the default GeneMANIA network weighting settings, with all available interaction data sources included. The GeneMANIA platform was accessed on 12 December 2025.

## 3. Results

**Figure 1:**
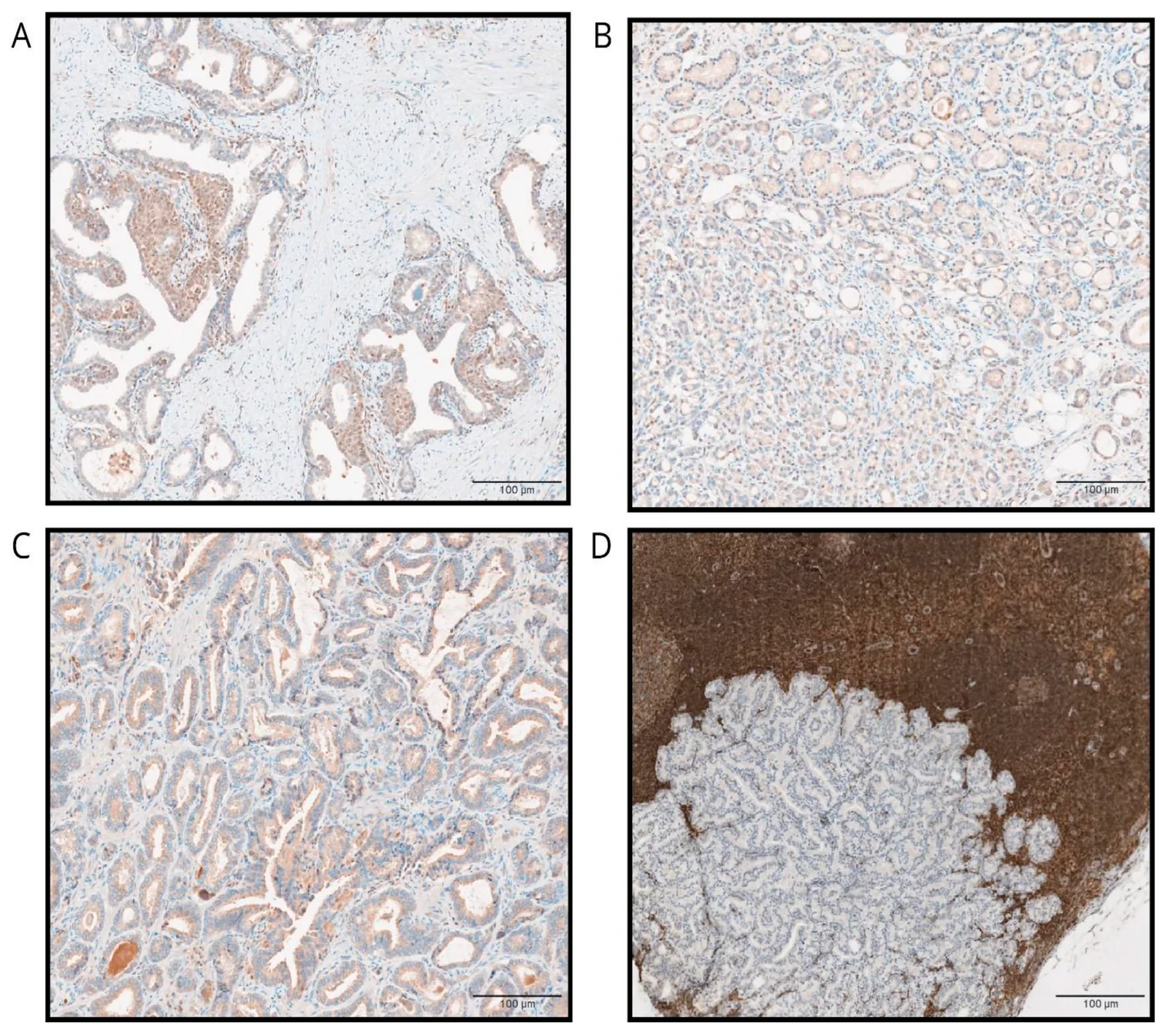
Representative immunohistochemical images of FGD3 expression in adjacent non-cancerous prostate tissue (**A**), prostate adenocarcinoma (PRAD) with low FGD3 expression (**B**), PRAD with high FGD3 expression (**C**), and lymph node metastasis (**D**) (×100).

Immunohistochemical analysis demonstrated cytoplasmic and membranous localization of FGD3 in prostate tissues. FGD3 expression was clearly detectable in control prostate tissue, whereas prostate cancer samples showed heterogeneous staining, with both low and high FGD3 expression observed among tumor cases. In lymph node metastases, a marked reduction in staining intensity was generally noted compared with the primary tumor and control tissues. Representative immunohistochemical images are shown in Figure 1.

Among the 185 prostate cancer samples analyzed, low FGD3 expression was identified in 91 cases (49.19%), while high FGD3 expression was observed in 94 cases (50.81%), according to the established H-score cut-off value of 170.

**Figure 2:**
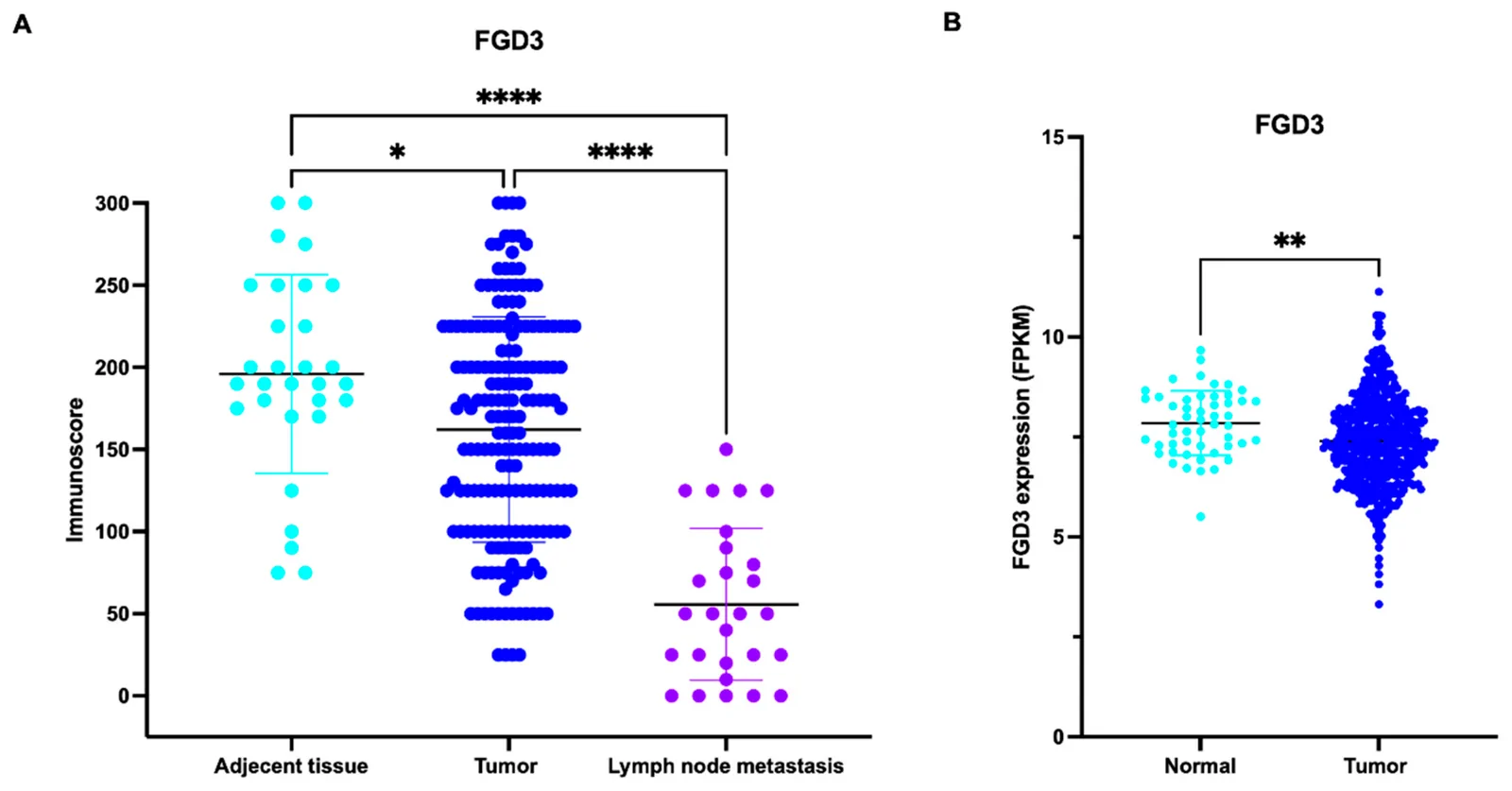
FGD3 expression in prostate cancer tissues compared with adjacent non-cancerous tissue and lymph node metastases. Boxplot graphs showing FGD3 protein expression in adjacent non-cancerous prostate tissue (n = 30), primary prostate cancer tissue (n = 185), and lymph node metastases (n = 27) in our cohort (**A**); and FGD3 mRNA expression levels in prostate cancer tissue and matched adjacent normal prostate tissue based on RNA-Seq data from the TCGA-PRAD cohort (**B**). The horizontal line indicates the median, and each dot represents an individual sample. Statistical significance was determined using non-parametric tests. * *p* < 0.05; ** *p* < 0.01; **** *p* < 0.0001.

In the subset of 27 patients for whom matched adjacent non-cancerous prostate tissue, primary prostate cancer tissue, and lymph node metastasis samples were available, FGD3 expression differed significantly among the three tissue types (Friedman test, χ^2^(2) = 36.96, *p* < 0.0001; Kendall’s W = 0.684). The median FGD3 H-score progressively decreased from 190.0 (IQR: 176.2–243.8) in adjacent non-cancerous prostate tissue to 170.0 (IQR: 100.0–225.0) in primary prostate cancer tissue and 50.0 (IQR: 22.5–85.0) in lymph node metastases. Post hoc pairwise comparisons using the Wilcoxon matched-pairs signed-rank test with Holm correction demonstrated significantly lower FGD3 expression in primary prostate cancer tissue compared with matched adjacent non-cancerous tissue (Holm-adjusted *p* = 0.0120). FGD3 expression was further markedly reduced in lymph node metastases compared with both matched primary prostate cancer tissue (Holm-adjusted *p* < 0.0001) and adjacent non-cancerous prostate tissue (Holm-adjusted *p* < 0.0001) (Figure 2A).

External validation using RNA-seq data from the TCGA-PRAD cohort confirmed a significant difference in FGD3 mRNA expression between normal prostate tissues and prostate cancer tissues. In the TCGA-PRAD cohort, FGD3 mRNA expression was significantly higher in non-cancerous prostate tissue than in prostate cancer tissue (median 7.820, IQR 7.285–8.441 vs. 7.339, IQR 6.629–8.124; Mann–Whitney U = 16,070.5, *p* = 0.0012). The rank-biserial correlation was 0.273 (95% CI: 0.134–0.408), indicating a modest difference in FGD3 expression between the two groups. FGD3 expression was observed in cancer cells and non-cancerous prostatic gland tissue (Figure 2B).

### 3.1. Expression of FGD3 and Its Clinicopathological Association

The associations between FGD3 expression and clinicopathological parameters of patients with prostate cancer are presented in Table 1. Among the 185 analyzed cases, low FGD3 expression was observed in 91 patients, whereas high FGD3 expression was detected in 94 patients.

FGD3 expression was significantly associated with Gleason score (*p* < 0.0001). High FGD3 expression was more frequently observed in tumors with lower Gleason scores, including Gleason scores 6 and 7, where high expression was detected in 84.2% and 67.6% of cases, respectively. In contrast, low FGD3 expression predominated in tumors with higher Gleason scores, particularly Gleason scores 8 and 9, where low expression was observed in 81.5% and 89.2% of cases, respectively.

A significant association was also found between FGD3 expression and Grade Group (*p* < 0.0001). High FGD3 expression was more common in Grade Groups 1–3, whereas low FGD3 expression was predominant in higher-grade tumors, especially Grade Group 4 and Grade Group 5, in which low FGD3 expression was observed in 77.8% and 89.2% of cases, respectively.

FGD3 expression was additionally associated with pathological T stage (*p* = 0.0385). High FGD3 expression was more frequently detected in pT2 tumors (58.9%), whereas low FGD3 expression was more common in pT3 tumors (53.7%) and was observed in all pT4 cases, although the number of pT4 cases was limited.

No significant associations were observed between FGD3 expression and patient age (*p* = 0.295), nodal status (*p* = 0.301), or PSA level (*p* = 0.187).

The association between FGD3 mRNA expression and clinicopathological parameters was additionally analyzed in the TCGA-PRAD cohort are presented in Table 2. A total of 490 patients with available clinical and transcriptomic data were included in this analysis. Based on the established cut-off value, low FGD3 expression was observed in 283 cases, whereas high FGD3 expression was identified in 207 cases.

FGD3 mRNA expression was significantly associated with tumor stage (*p* = 0.0231). High FGD3 expression was more frequently observed in tumors classified as T3–T4 compared with T2 tumors. Among patients with T2 tumors, high FGD3 expression was detected in 66 cases (35.5%), whereas in the T3–T4 group, high FGD3 expression was observed in 137 cases (46.1%).

A significant association was also found between FGD3 expression and lymph node invasion (*p* = 0.0114). High FGD3 expression was detected in 44 patients with lymph node involvement (56.4%) and in 137 patients without lymph node invasion (40.4%).

No significant associations were observed between FGD3 mRNA expression and age (*p* = 0.4392), race (*p* = 0.8515), or progression status (*p* = 0.2777). Specifically, high FGD3 expression was found in 40.9% of patients younger than 65 years and in 44.8% of patients aged 65 years or older. In terms of progression, high FGD3 expression was detected in 41.0% of patients without progression and in 47.8% of patients with progression.

### 3.2. Survival Analysis

**Figure 3:**
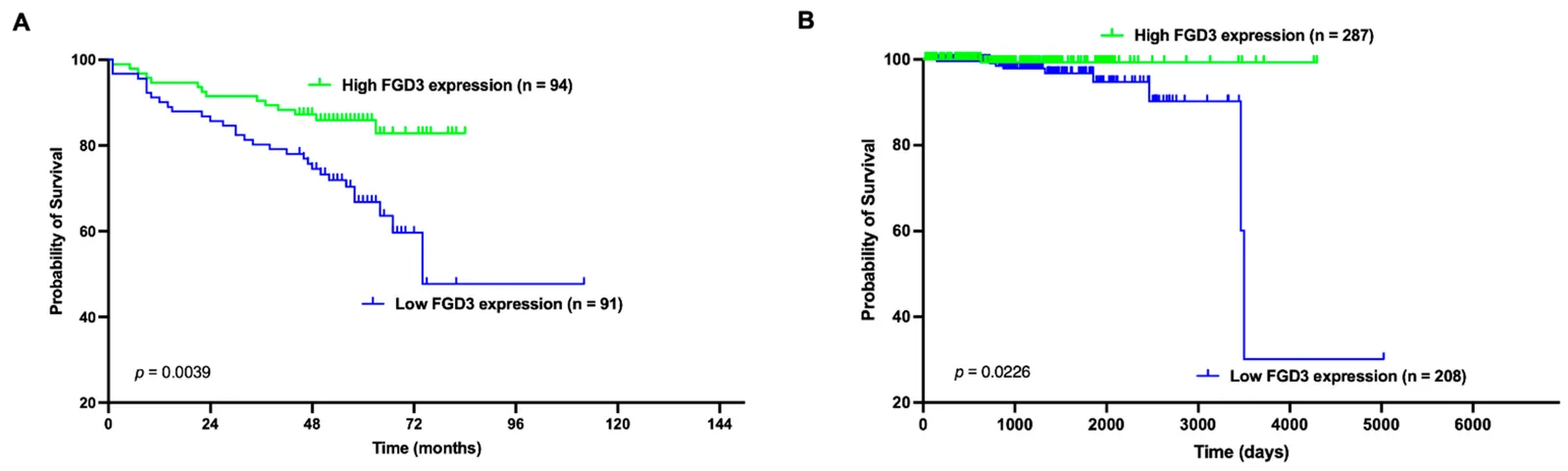
Log-rank test for OS of PRAD patients based on FGD3 expression in our cohort (**A**) and TCGA (**B**).

Kaplan–Meier survival analysis was performed to evaluate the prognostic significance of FGD3 expression in prostate cancer patients (Figure 3). In our cohort, patients with low FGD3 expression had significantly shorter overall survival (OS) compared with patients showing high FGD3 expression (*p* = 0.0039, log-rank test). The median OS in the low FGD3 expression group was 74 months, whereas the median OS in the high FGD3 expression group was not reached. The hazard ratio for the low versus high FGD3 expression group was 2.451 (95% CI: 1.365–4.400).

The prognostic value of FGD3 was further assessed using RNA-seq data from the TCGA-PRAD cohort. Kaplan–Meier analysis also demonstrated a significant association between FGD3 mRNA expression and OS in this external dataset (*p* = 0.0226, log-rank test). Patients with low FGD3 mRNA expression showed shorter survival compared with those with high FGD3 expression. The median OS was 3502 days in the low-expression group, whereas it was not reached in the high-expression group. The hazard ratio for low versus high FGD3 expression was 7.396 (95% CI: 2.128–25.70).

D’Amico risk classification for PCa was calculated with the Evidencio Medical tool [10].

A total of 128 patients were classified as a high risk, and 44 patients were classified as intermediate risk and low risk, 13 patients according to D’Amico’s score (Table 3).

As presented in Table 4, univariate Cox regression analysis showed that high FGD3 expression was significantly associated with better overall survival (HR 0.41, 95% CI 0.22–0.77, *p* = 0.005). In addition, pN status (HR 2.05, 95% CI 1.04–4.06, *p* = 0.039) and Grade Group 4–5 (HR 2.07, 95% CI 1.15–3.72, *p* = 0.015) were significantly associated with OS. In multivariable analysis, high FGD3 expression showed a favorable prognostic trend, although it did not reach statistical significance (HR 0.50, 95% CI 0.24–1.07, *p* = 0.073). No variable remained an independent prognostic factor for OS in the multivariable model.

As presented in Table 5, univariate Cox regression analysis in the TCGA-PRAD cohort showed that progression was significantly associated with poorer OS (HR 6.37, 95% CI 1.56–25.98, *p* = 0.010). High FGD3 expression showed a favorable prognostic trend compared with low FGD3 expression, although the association did not reach statistical significance (HR 0.13, 95% CI 0.02–1.01, *p* = 0.051). Age ≥ 65 years (HR 2.88, 95% CI 0.81–10.22, *p* = 0.101), tumor stage T3–T4 (HR 3.31, 95% CI 0.62–17.79, *p* = 0.163), and lymph node invasion (HR 3.55, 95% CI 0.79–16.04, *p* = 0.100) were not significantly associated with OS in univariate analysis. In multivariable Cox regression analysis, age ≥ 65 years (HR 4.59, 95% CI 1.04–20.35, *p* = 0.045) and progression (HR 5.49, 95% CI 1.19–25.26, *p* = 0.029) remained independent prognostic factors for OS. High FGD3 expression maintained a favorable prognostic trend, but without statistical significance (HR 0.15, 95% CI 0.02–1.25, *p* = 0.079). Lymph node invasion also showed a non-significant trend toward poorer OS (HR 4.43, 95% CI 0.90–21.90, *p* = 0.068).

### 3.3. FGD3 Interaction Network and Functional Enrichment Analysis

**Figure 4:**
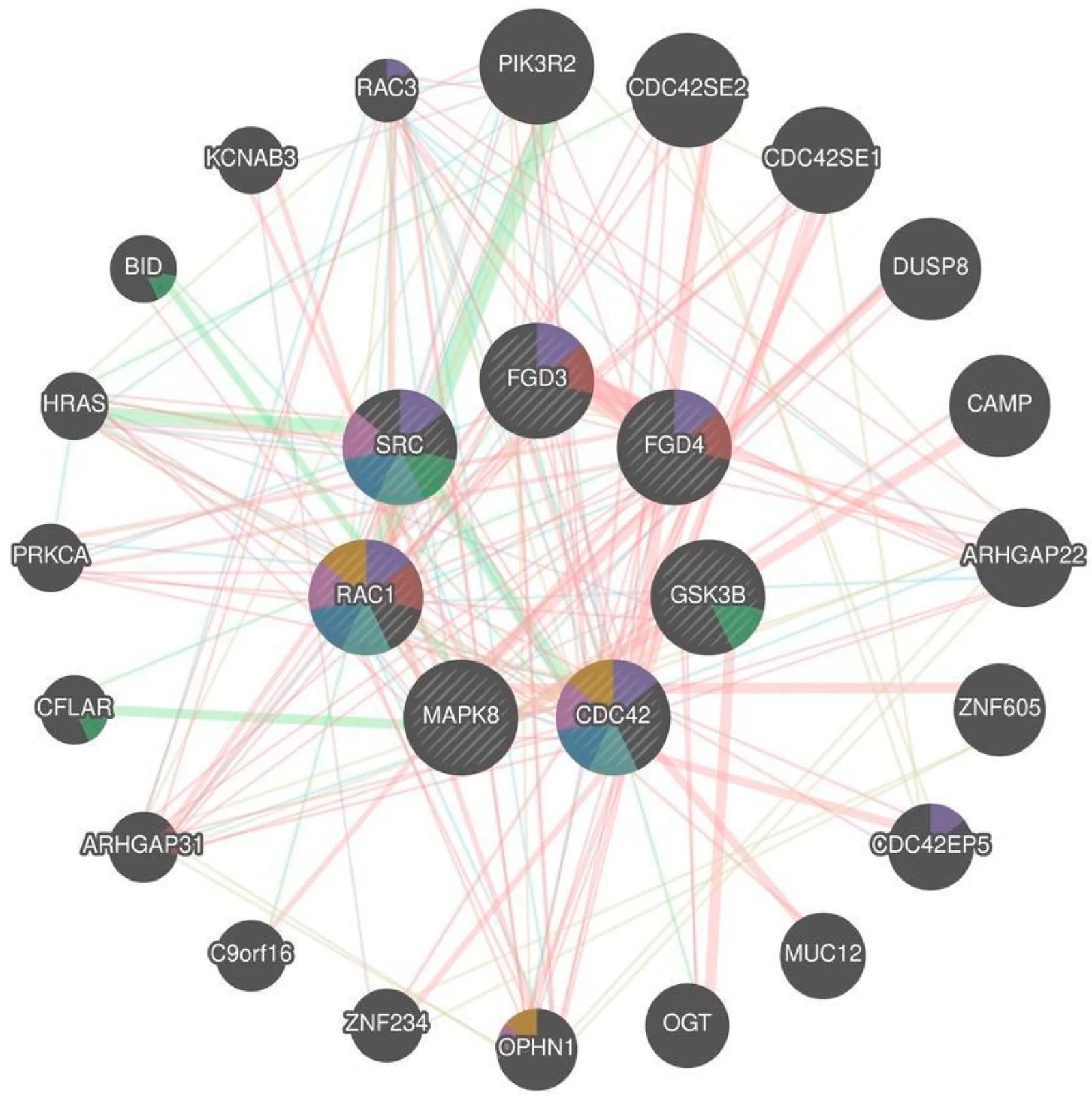
Potential network pathway for the top genes connected with FGD3 and PRAD. The nodes and edges are retrieved from the GeneMANIA database and plotted using Cytoscape software (version 3.5.3).

The Protein–Protein Interaction (PPI) network was initially constructed by importing the 27 genes: FGD3, FGD4, GSK3B, CDC42, MAPK8, RAC1, SRC-7, OPHN1, ZNF234, C9orf16, ARHGAP31, CFLAR, PRKCA, HRAS, BID, KCNAB3, RAC3, PIK3R2, CDC42SE2, CDC42SE1, DUSP8, CAMP, ARHGAP22, ZNF605, CDC42EP5, MUC12, and OGT (Figure 4). Functional enrichment analysis was then applied to this set of proteins to provide insights into their potential roles in PCa. The enriched functional terms related to the queried genes mainly included regulation of cell morphogenesis, cell leading edge, regulation of apoptotic signaling pathway, lymphocyte costimulation, positive regulation of lamellipodium organization, regulation of endocytosis, and Rho protein signal transduction. Nodes connected by pink edges presented direct physical interactions (58.82%), nodes connected by green edges showed genetic interactions (17.64%), and nodes linked via blue edges shared the same signal pathway (8.02%).

## 4. Discussion

The incidence of PRAD has been notably increasing; it is the second most prevalent malignancy among males. Although PSA testing, digital rectal examination, multiparametric imaging, histopathological grading, and pathological stage remain central to diagnosis and risk stratification, these parameters do not fully explain the biology of PRAD. Some tumors remain indolent for years, whereas others progress rapidly despite apparently localized disease. This discrepancy supports the continued search for molecular markers that may complement established clinicopathological variables and improve biological risk assessment.

Our study evaluated the expression and prognostic relevance of FGD3 in prostate adenocarcinoma and demonstrated a consistent association between reduced FGD3 expression and adverse tumor characteristics. FGD3 expression was significantly lower in primary PCa than in adjacent non-cancerous prostate tissue and showed a further marked reduction in lymph node metastases. Within the primary tumor cohort, low FGD3 expression was strongly associated with higher Gleason score and higher ISUP Grade Group. It was also more frequent in tumors with advanced pathological T stage. In addition, patients with low FGD3 expression had significantly shorter overall survival. Together, these findings suggest that loss of FGD3 expression accompanies increasing histopathological aggressiveness and disease progression in PCa.

The association with tumor grade was particularly pronounced. High FGD3 expression was present in Gleason scores 6 and 7 tumors, whereas low expression was present in 81.5% of Gleason 8 and 89.2% of Gleason 9 tumors. A similar pattern was observed across ISUP Grade Groups, with low FGD3 expression present in 77.8% of Grade Group 4 and 89.2% of Grade Group 5 tumors. FGD3 expression was also associated with the pathological T stage, being more frequently preserved in pT2 disease and reduced in pT3 tumors. All three pT4 tumors showed low expression, although the small number of cases precludes further interpretation. Consistent with these findings, most patients classified as high risk according to the D’Amico system belonged to the FGD3-low group. This observation should, however, be regarded as descriptive because a formal association test was not performed. In contrast, no significant association was found between FGD3 expression and age, PSA level, or nodal status in the primary tumor cohort. Thus, the strongest associations observed in our study concern histological differentiation and local tumor advancement rather than conventional demographic or biochemical parameters.

The marked reduction of FGD3 expression in lymph node metastases represents another important finding. Median FGD3 H-score decreased from 190 in adjacent non-cancerous prostate tissue to 170 in primary tumors and to 50 in lymph node metastases. Although low FGD3 expression in the primary tumor was not significantly associated with pN status, metastatic deposits themselves demonstrated substantially lower expression than both primary tumors and non-cancerous tissue. These findings suggest that reduced FGD3 expression may accompany phenotypic changes occurring during tumor progression and metastatic dissemination. Whether FGD3 downregulation contributes directly to this process or instead reflects selection of more poorly differentiated tumor-cell populations cannot be determined from the present study.

The known biological functions of FGD3 provide a plausible framework for these observations. FGD3 belongs to the Rho guanine nucleotide exchange factor family and contains DH, PH, and FYVE domains involved in the spatial regulation of Rho-family signaling and membrane-associated processes [5,14]. Rho-family GTPases regulate cytoskeletal organization, cell polarity, migration, endocytosis, and cell-cycle-related processes [5,15]. These pathways are directly relevant to cancer invasion and metastasis. In the present PPI analysis, FGD3-associated networks were enriched for regulation of cell morphogenesis, lamellipodium organization, endocytosis, apoptotic signaling, and Rho protein signal transduction, further supporting a similar pathway between FGD3-related signaling and invasive tumor behavior.

The FGD3-associated network contained several regulators of Rho-family signaling, including CDC42, RAC1, and RAC3, and was enriched for biological processes involving cell morphogenesis, the cell leading edge, lamellipodium organization, endocytosis, and Rho protein signal transduction [14,15]. Lamellipodia and the leading edge are central structures involved in actin-dependent cell movement and dynamic interactions with the extracellular environment. The convergence of these pathways is therefore compatible with a potential role of FGD3 in regulating cellular architecture and motility. Importantly, the network analysis does not establish that loss of FGD3 directly activates these pathways or causes metastatic behavior.

The Kaplan–Meier analyses further support the potential prognostic relevance of FGD3. Patients with low FGD3 protein expression in our cohort had significantly shorter overall survival than those with high expression. In univariable Cox regression, high FGD3 expression was associated with a lower risk of death, together with nodal status and higher Grade Group. However, the association between FGD3 and survival did not remain statistically significant after multivariable adjustment. Whether FGD3 provides prognostic information beyond conventional clinicopathological variables requires validation in larger independent cohorts.

Our findings also appear consistent with the broader literature on FGD3 in cancers. Willis et al. identified high FGD3 expression as a marker of a favorable outcome across several malignancies [7]. In breast cancer, both transcriptomic and immunohistochemical studies have associated preserved or high FGD3 expression with more favorable clinicopathological characteristics and improved survival [9,10]. Similar favorable prognostic associations have been reported in head and neck squamous cell carcinoma [8], while FGD3 has also been described as a protective component of prognostic signatures in stage II/III colorectal cancer and lung adenocarcinoma [11,12]. Although the biological role of FGD3 likely depends on tumor context, these observations collectively support a recurring pattern in which low FGD3 expression is associated with a more aggressive phenotype or an unfavorable clinical outcome. The present study extends this observation to prostate adenocarcinoma.

Several limitations should be acknowledged. This was a retrospective, single-center study of patients undergoing RP, with an overrepresentation of locally advanced and higher-risk disease. Consequently, the cohort does not reflect the full biological spectrum of PCa, particularly low-risk tumors managed without radical treatment. The number of lymph node metastasis samples was limited. Furthermore, overall survival was used as the survival endpoint and may be influenced by age, comorbidities, and causes of death unrelated to PCa. The H-score cut-off was optimized within the study cohort and therefore requires independent validation. TCGA validation was performed at the mRNA rather than protein level and is subject to the limitations of bulk transcriptomic analysis. Functional experiments were not performed, preventing conclusions regarding whether FGD3 loss has a causal role in tumor invasion or metastasis.

Overall, reduced FGD3 expression was associated with more aggressive PCa. It was linked to higher tumor grade, more advanced local stage, lower expression in lymph node metastases, and shorter overall survival. However, FGD3 was not an independent prognostic factor in multivariable analysis. Its prognostic value, therefore, requires further validation. Future studies should also clarify whether loss of FGD3 contributes directly to PCa progression or simply reflects a more aggressive tumor phenotype.

## 5. Conclusions

Our findings indicate that reduced FGD3 expression has adverse prognostic significance in PCa. This observation is consistent with studies in other cancers, including breast, head and neck, colorectal, and lung cancers, in which preserved or high FGD3 expression has generally been associated with more favorable outcomes. In our cohort, FGD3 expression was significantly lower in tumor tissue than in adjacent non-cancerous prostate tissue and showed the most marked reduction in lymph node metastases. These findings may reflect differences in Rho-family/Rac-related signaling, including c-Src-mediated pathways, as well as the specific hormonal and stromal microenvironment of the prostate. A notable limitation of our cohort is the relatively advanced age of the patients and the use of overall survival as the endpoint. Therefore, the survival data are not disease-specific. PCa classified as grade group 1, Gleason score 3 + 3, and organ-confined disease generally have a favorable prognosis. In clinical practice, comparable low-risk cases are often managed with active surveillance or watchful waiting in selected patients, rather than immediate radical treatment. Such indolent tumors represent a substantial proportion of PCa diagnoses, whereas our cohort had more aggressive forms of PCa and was characterized by a higher mortality rate. The association between low FGD3 expression and worse survival in PCa supports further investigation. In our opinion, the implementation of FGD3 as a single prognostic marker for PCa is not justified at this stage. Further studies are needed to clarify its biological role and its correlations with established clinicopathological and molecular factors.

## Figures and Tables

**Table 1:** Association between expression of FGD3 and clinical characteristics in our cohort.

Characteristic	Category	All (N = 185)	FGD3 Low (N = 91)	FGD3 High (N = 94)	*p* Value	FDR-Adjusted *p* Value (Benjamini–Hochberg)	Cramér’s V
Age (years)	<65	75 (40.5)	33 (44.0)	42 (56.0)	0.295	0.301	0.086
	≥65	110 (59.5)	58 (52.7)	52 (47.3)			
Gleason score	6	19 (10.3)	3 (15.8)	16 (84.2)	**<0.0001**	**<0.0001**	0.545
	7	102 (55.1)	33 (32.4)	69 (67.6)			
	8	27 (14.6)	22 (81.5)	5 (18.5)			
	9	37 (20.0)	33 (89.2)	4 (10.8)			
Grade Group	1	19 (10.3)	3 (15.8)	16 (84.2)	**<0.0001**	**<0.0001**	0.528
	2	68 (36.8)	24 (35.3)	44 (64.7)			
	3	34 (18.4)	10 (29.4)	24 (70.6)			
	4	27 (14.6)	21 (77.8)	6 (22.2)			
	5	37 (20.0)	33 (89.2)	4 (10.8)			
pT	T2	74 (40.0)	30 (40.5)	44 (59.5)	**0.0385**	0.0770	0.183
	T3	108 (58.4)	58 (53.7)	50 (46.3)			
	T4	3 (1.6)	3 (100.0)	0 (0.0)			
pN	N0	158 (85.4)	75 (47.5)	83 (52.5)	0.301	0.301	0.083
	N1	27 (14.6)	16 (59.3)	11 (40.7)			
PSA (ng/mL)	<10	97 (52.4)	43 (44.3)	54 (55.7)	0.187	0.280	0.102
	≥10	88 (47.6)	48 (54.5)	40 (45.5)			

Significant *p*-values (*p* < 0.05) are indicated in bold.

**Table 2:** Association between expression of FGD3 and clinical characteristics in the TCGA-PRAD cohort.

Characteristic	Category	All (N = 490 *)	FGD3 Low (N = 283)	FGD3 High (N = 207)	*p* Value	FDR-Adjusted *p* Value (Benjamini–Hochberg)	Cramér’s V
Age (years) (n = 490)	<65	325 (66.3%)	192 (59.1%)	133 (40.9%)	0.4392	0.5490	0.038
	≥65	165 (33.7%)	91 (55.2%)	74 (44.8%)			
Race (n = 156)	White	147 (94.2%)	98 (66.7%)	49 (33.3%)	1.0000	1.0000	0.045
	Black or African-American	7 (4.5%)	5 (71.4%)	2 (28.6%)			
	Asian	2 (1.3%)	1 (50.0%)	1 (50.0%)			
Tumor stage (n = 483)	T2	186 (38.5%)	120 (64.5%)	66 (35.5%)	**0.0231**	0.0578	0.105
	T3–T4	297 (61.5%)	160 (53.9%)	137 (46.1%)			
Lymph nodes invasion (n = 417)	N0	339 (81.3%)	202 (59.6%)	137 (40.4%)	**0.0114**	0.0569	0.126
	N1–N3	78 (18.7%)	34 (43.6%)	44 (56.4%)			
Progression (n = 490)	No progression	398 (81.2%)	235 (59.0%)	163 (41.0%)	0.2430	0.4049	0.054
	Progression	92 (18.8%)	48 (52.2%)	44 (47.8%)			

* The number of evaluable cases varies because of missing data in the TCGA database: age, n = 490; race, n = 156; tumor stage, n = 483; lymph node invasion, n = 417; and progression, n = 490. Percentages in the all column were calculated using the number of evaluable cases for each characteristic as the denominator. Significant *p*-values (*p* < 0.05) are indicated in bold.

**Table 3:** D’Amico risk classification for prostate cancer (calculated with Evidencio Medical Decision Support).

D’Amico Risk Group	PSA (ng/mL)	Gleason Score	Stage Criterion	Total n (%)	FGD3 Low n (%)	FGD3 High n (%)
Low risk	<10	≤6	T1–T2a	13 (7.0)	2 (2.2)	11 (11.7)
Intermediate risk	10–20	7	T2b	44 (23.8)	14 (15.4)	30 (31.9)
High risk	>20	8–10	≥T2c/T3–T4	128 (69.2)	75 (82.4)	53 (56.4)

**Table 4:** The univariable and multivariable Cox regression models in PRAD patients (our cohort, n = 185).

Variable	Univariate HR (95% CI)	Univariate *p* Value	Multivariable HR (95% CI)	Multivariable *p* Value
FGD3 high vs. low	0.41 (0.22–0.77)	**0.005**	0.50 (0.24–1.07)	0.073
Age ≥ 65 vs. <65	1.65 (0.88–3.11)	0.120	–	–
PSA ≥ 10 vs. <10	1.32 (0.74–2.39)	0.349	–	–
pN N1 vs. N0	2.05 (1.04–4.06)	**0.039**	1.81 (0.91–3.60)	0.092
pT T3–T4 vs. T1–T2	1.47 (0.78–2.77)	0.230	–	–
Grade Group 4–5 vs. 1–3	2.07 (1.15–3.72)	**0.015**	1.34 (0.66–2.70)	0.418

Abbreviations: CI—confidence interval; HR—hazard ratio. Significant *p*-values (*p* < 0.05) are indicated in bold.

**Table 5:** The univariable and multivariable Cox regression models in PRAD patients (TCGA-PRAD cohort, n = 190).

Variable	Univariate HR (95% CI)	Univariate *p* Value	Multivariable HR (95% CI)	Multivariable *p* Value
FGD3 high vs. low	0.13 (0.02–1.01)	0.051	0.15 (0.02–1.25)	0.079
Age ≥ 65 vs. <65	2.88 (0.81–10.22)	0.101	4.59 (1.04–20.35)	**0.045**
Tumor stage T3–T4 vs. T2	3.31 (0.62–17.79)	0.163	–	–
Lymph node invasion N1–N3 vs. N0	3.55 (0.79–16.04)	0.100	4.43 (0.90–21.90)	0.068
Progression vs. no progression	6.37 (1.56–25.98)	**0.010**	5.49 (1.19–25.26)	**0.029**

Abbreviations: CI—confidence interval; HR—hazard ratio. Significant *p*-values (*p* < 0.05) are indicated in bold.

## Data Availability

The datasets generated and analyzed during the current study can be obtained from the corresponding author upon request.

## Abbreviations

aPKC	Atypical Protein Kinase C
ARHGAP22	Rho GTPase Activating Protein 22
ARHGAP31	Rho GTPase Activating Protein 31
BID	BH3 Interacting Domain Death Agonist
C9orf16	Chromosome 9 Open Reading Frame 16
CAMP	Cathelicidin Antimicrobial Peptide
CDC42	Cell Division Cycle 42
CDC42EP5	CDC42 Effector Protein 5
CDC42SE1	CDC42 Small Effector Protein 1
CDC42SE2	CDC42 Small Effector Protein 2
CFLAR	CASP8 and FADD-Like Apoptosis Regulator
CI	Confidence Interval
c-MYC	Cellular Myelocytomatosis
cSrc	Cellular Sarcoma kinase
Dbl	Diffuse B-cell lymphoma
DH	Diffuse B-cell lymphoma Homology
DSGIDS	Conserved Destruction Motif
DUSP8	Dual Specificity Phosphatase 8
E2	Estradiol
ERα	Estrogen Receptor Alpha
ERβ	Estrogen Receptor Beta
ESR1	Estrogen Receptor 1
FFPE	Formalin-Fixed Paraffin-Embedded
FGD3	Faciogenital Dysplasia 3
FGD4	Faciogenital Dysplasia 4
GDP	Guanosine Diphosphate
GPER	G Protein-Coupled Estrogen Receptor
GSK3B	Glycogen Synthase Kinase 3 Beta
GSK-3β	Glycogen Synthase Kinase 3 Beta
H&E	Hematoxylin and Eosin
HLA-A	Human Leukocyte Antigen A
HLA-B	Human Leukocyte Antigen B
HR	Hazard Ratio
HRAS	Harvey Rat Sarcoma viral oncogene homolog
IHC	Immunohistochemistry
IκBs	Inhibitor of Kappa B
JNK	c-Jun N-terminal Kinase
KCNAB3	Potassium Voltage-Gated Channel Subfamily A Member 3
LNM	Lymph Node Metastasis
MAPK8	Mitogen-Activated Protein Kinase 8
mRNA	Messenger RNA
MUC12	Mucin 12
OGT	O-Linked N-Acetylglucosamine Transferase
OPHN1	Oligophrenin 1
OS	Overall Survival
PAK1	p21-activated Kinase 1
PCa	Prostate cancer
PI3P	Phosphatidylinositol 3-phosphate
PIK3R2	Phosphoinositide-3-Kinase Regulatory Subunit 2
pM	Pathological Metastasis stage
pN	Pathological Node stage
PPI	Protein–Protein Interaction
PRAD	Prostate adenocarcinoma
pT	Pathological Tumor stage
RAC1	Ras-related C3 botulinum toxin substrate 1
RAC3	Ras-related C3 botulinum toxin substrate 3
Ran	Ras-related Nuclear Protein
Rho	Ras Homolog
RhoGEFs	Rho Guanine Nucleotide Exchange Factors
RT-qPCR	Real-Time Quantitative Polymerase Chain Reaction
Sar1	Secretion Associated Ras-related GTPase 1A
SRC	Cellular Sarcoma kinase
SRC-7	Cellular Sarcoma kinase 7
SUSD3	Sushi Domain-containing 3
TMAs	Tissue Macroarrays
TMPRSS2-ERG	Transmembrane Protease, Serine 2–ETS-related Gene
TNM	Tumor, Node, Metastasis
UALCAN	University of Alabama at Birmingham CANcer data analysis Portal
ZNF234	Zinc Finger Protein 234
ZNF605	Zinc Finger Protein 605
